# Supplementary material for: Specifics of Young Gastric Cancer Patients: A Population-Based Analysis of 46,110 Patients with Gastric Cancer from the German Clinical Cancer Registry Group
Source: Cancers (Basel). 2022 Nov 30;14(23):5927. doi: 10.3390/cancers14235927 (PMC9739447; doi:10.3390/cancers14235927)
Supplement: Supplementary file 1 [file cancers-14-05927-s001.zip › cancers-2071156-supplementary.pdf]

Supplementary Material

Table S1. Details on modified study parameters from basis dataset.

Variablenname	Feldinhalt
source	Data orgin, registry
PATID	Intern patienidentification, Pseudonym
Date of birth	Date of birth
date_alive	Date of last known “alive status”
Date of death	Date of death
sex	sex
Zip code	Zip code
Date of diagnosis	Date of diagnosis
localization_ICDO3	localization according to ICD-O3
diagnosis_ICD10	ICD-10 Code
histology	Histology key
Grading	Grading
cT	cT-stage
cN	cN-stage
cM	cM-stage
state_clinical	Clinical stage of tumor
pT	pT-stage
pN	pN-stage
pM	pM-stage
stage_patho	Pathological stage of tumor
ypT	ypT-stage
ypN	ypN-stage
ypM	ypM-stage
stage_neo_patho	Stage of tumor after neoadjuvant therapy
LN_infested	Infested amount of lymph nodes
LN_examined	Examined amount of lymph nodes
OP_date	Date of primary relevant surgery
OP_intention	Intention of surgery
OPS1	OPS-code / ICPM-code. Just for primary relevant surgery
OP_local	Code for primary relevant surgery group if OPS was unkown
OP_complication	Complication of surgery

Lymphadenectomy	Extent of lymphadenectomy
R_classification_local	Resection of Tumor (R0, R1, R2)
Radiatio_start	Date of radiation start
Radiatio	Specification if radiatio was intendend and took place
Radiatio_neoadjuvant	Specification if neoadjuvant radiatio was intendend and took place
Radiatio_adjuvant	Specification if adjuvant radiatio was intendend and took place
Radiatio_palliativ	Specification if palliative radiatio was intendend and took place
Radiochemo_definitiv	Specification if definite radiatio was intendend and took place
Chemo_start	Date of chemotherapy start
Chemo_perioperativ	Specification if perioperative chemotherapy was intendend and took place
Chemotherapie	Specification if chemotherapy was intendend and took place
Chemo_neoadjuvant	Specification if neoadjuvant chemotherapy was intendend and took place
Chemo_adjuvant	Specification if adjuvant chemotherapy was intendend and took place
Chemo_palliativ	Specification if palliative chemotherapy was intendend and took place
date_locoregional_recurrence	Date of first local recurrence or locoregional infestet lymph node recurrence
date_metastasis	Date of first metastasis (synchronous or metachronous)
localization metastasis	Location of metastasis
date_event	Date of first unspecific metastasis event (no further data for metastatis event)
date_freeoftumor	last date of tumor free status
Hospital_Practice_Nr	Freely selectable and anonymous key number for main treatment facility (primary therapy, clinic or practice, independent of recognition as certified center)
Zentrumsbehandlung	Center treatment yes/no
date_first_hepatic_metastasis	Date of first liver metastasis, synchronous or metachronous with the diagnosis of carcinoma
date_OP_hepatic_metastasis	Date of surgery for the first liver metastasis
Type_OP_Lebermetastasen	Type of surgery for liver metastases
tumorconference_Datum	Date of pre-therapeutic presentation in an interdisciplinary tumor conference
ASA_Classification	ASA Classification
Type_Op	Elective or emergency surgery
General_condition	ECOG or Karnofsky-Index
ProtocolChemo_perioperative	Protocol of perioperative chemotherapy
ProtocolChemotherapy	Chemotherapy protocol
ProtocolChemo_neoadjuvant	Neoadjuvant chemotherapy protocol
ProtocolChemo_adjuvant	Adjuvant chemotherapy protocol
ProtocolChemo_palliativ	Palliative chemotherapy protocol
